# Fabrication and Characteristics of a SOI Three-Axis Acceleration Sensor Based on MEMS Technology

**DOI:** 10.3390/mi10040238

**Published:** 2019-04-09

**Authors:** Xiaofeng Zhao, Ying Wang, Dianzhong Wen

**Affiliations:** The Key Laboratory of Electronics Engineering, College of Heilongjiang Province, Heilongjiang University, Harbin 150080, China; 2181212@s.hlju.edu.cn (Y.W.); wendianzhong@hlju.edu.cn (D.W.)

**Keywords:** three-axis acceleration sensor, MEMS technology, sensitivity, L-shaped beam

## Abstract

A silicon-on-insulator (SOI) piezoresistive three-axis acceleration sensor, consisting of four L-shaped beams, two intermediate double beams, two masses, and twelve piezoresistors, was presented in this work. To detect the acceleration vector (*a_x_*, *a_y_*, and *a_z_*) along three directions, twelve piezoresistors were designed on four L-shaped beams and two intermediate beams to form three detecting Wheatstone bridges. A sensitive element simulation model was built using ANSYS finite element simulation software to investigate the cross-interference of sensitivity for the proposed sensor. Based on that, the sensor chip was fabricated on a SOI wafer by using microelectromechanical system (MEMS) technology and packaged on a printed circuit board (PCB). At room temperature and *V*_DD_ = 5.0 V, the sensitivities of the sensor along *x*-axis, *y*-axis, and *z*-axis were 0.255 mV/g, 0.131 mV/g, and 0.404 mV/g, respectively. The experimental results show that the proposed sensor can realize the detection of acceleration along three directions.

## 1. Introduction

Accelerometers have been used in many different fields, such as automotive industry, aviation and national security, aerospace engineering, biological engineering, etc. [1]. The main sensing mechanisms to convert acceleration into electrical signals include piezoresistive, capacitive, piezoelectric and resonant types, etc. Nevertheless, piezoresistive technique among of them has been attracted more attention due to its simple structures design and read out circuits, good direct current (DC) response, high sensitivity, linearity, and reliability as well as low cost. In 1979, Roylance et al. proposed a piezoresistive microsilicon accelerometer for the first time [2]. In addition, with the development of microelectromechanical system (MEMS) technology, acceleration sensors have been widely used in the field of inertial systems to test the acceleration of moving object [3,4,5]. Up to date, the three-axis acceleration sensor has realized the measurements of the velocity and posture for moving objects including unmanned aerial vehicle, gravity gradiometer, wearable acceleration sensor for monitoring human movement behavior, etc. [6,7]. Due to the extensive applications in many different fields, increasing demands for detection has triggered a particular research attention to improve the properties of three-axis acceleration sensor, such as miniaturization, high sensitivity, good consistency and low cross-interference of sensitivity, etc. For example, in 2011, Hsieh et al. designed a three-axis piezoresistive accelerometer with a stress isolation guard-ring structure, a low disturbance of environment and a big sensitivity range of 0.127 to 0.177 mV/(g·V) [8]. In 2016, Xu et al. fabricated a novel piezoresistive accelerometer with axially stressed sensing beams, not only improving the sensitivity and the resonant frequency at a supply voltage of 3.0 V, but also reducing the cross-axis sensitivity along *x*-axis and *z*-axis by less than 4.875 × 10^−6^ mV/g and 4.425 × 10^−6^ mV/g, respectively [9]. Thereafter, in 2017, Jung et al. proposed a monolithic piezoresistive high-g (20000 g) three-axis accelerometer with a single proof mass suspended using thin eight beams, achieving sensitivities of 0.243 mV/g, 0.131 mV/g, and 0.307 mV/g along the *x*-axis, *y*-axis and *z*-axis at a supply voltage of 5.0 V, respectively [10]. Meanwhile, Wang et al. presented a high-performance piezoresistive micro-accelerometer based on slot etching in an eight-beam structure to detect the vibration of a high speed spindle, improve the sensitivity and the natural frequency, as well as realize an average sensitivity of 0.785 mV/g at a supply voltage of 5.0 V [11]. In 2018, Marco et al. proposed a piezoresistive accelerometer based on a progressive moment of inertia (MMI) increment of the sensor proof mass in three-axis head injuries monitoring, obviously enhancing the sensitivity of the optimized structure along the *z*-axis up to 0.22 mV/g and obtaining low cross-interference less than 1% F.S. [12]. Meanwhile, Han et al. proposed a low cross-axis sensitivity piezoresistive accelerometer based on masked–maskless wet etching, which consisted of a proof mass, eight supporting beams, and four sensing beams, and achieved cross-axis sensitivities along *x*-axis and *y*-axis of 1.67% and 0.82%, respectively [13]. As the characteristics of the sensor are closely related to the sensing structure and the sensitive element of sensor, currently available methods have been adopted to improve the sensitivity and reduce the cross-interference, including modifying structure and selecting novel sensitive materials. 

In this paper, a silicon-on-insulator (SOI) three-axis acceleration sensor with four L-shaped beams, intermediate double beams, and two masses was presented. To detect the acceleration vector (*a_x_*, *a_y_*, and *a_z_*) along three directions and reduce the size of the chip, a basic structure of sensor was designed by using MEMS technology, and the corresponding working principle was investigated. Meanwhile, in order to reduce the cross-interference of sensitivity, how the sensitive element influences the cross-interference of sensitivity was analyzed by using ANSYS finite element simulation software. Based on that, the chip was fabricated on the SOI wafer by using MEMS technology and the thicknesses of cantilever beams can be effectively controlled, avoiding the effects of beams’ thickness on the sensitive characteristics. The study on the proposed sensor provides a new strategy for fabricating three-axis acceleration sensor to detect the acceleration vector. 

## 2. Basic Structure and Sensing Principle 

### 2.1. Basic Structure

**Figure 1:**
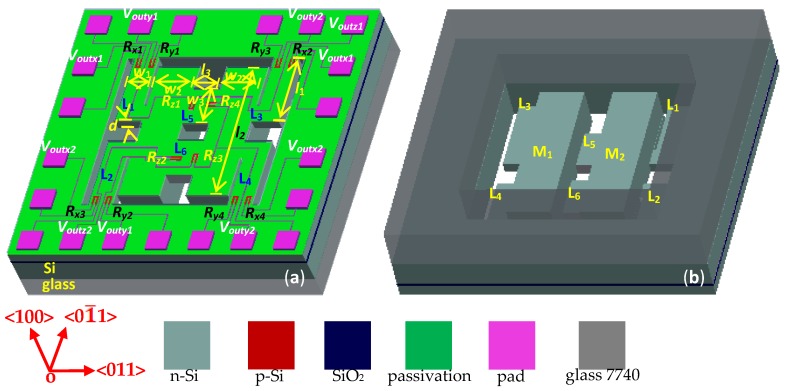
Basic structure of the silicon-on-insulator (SOI) three-axis acceleration sensor: (**a**) top view and (**b**) back view.

To easily release the structure of beams and better control the thickness of the beams by using the self-stop technology of inductively-coupled plasma (ICP) etching technology, a SOI wafer was utilized as a substrate of the proposed three-axis acceleration sensor. Figure 1a,b show the top and back views of the SOI three-axis acceleration sensor, respectively. The chip is composed of an elastic structure and a piezo-sensitive element as shown in Figure 1a, where the elastic structure includes four L-shaped beams (L_1_, L_2_, L_3_, and L_4_) and an intermediate double beam (L_5_ and L_6_). *l*_1_ (1200 μm) and *w*_1_ (200 μm) are the length and the width of the L-shaped beams for the proposed sensor, respectively. *l*_3_ (300 μm) and *w*_3_ (150 μm) are the length and the width of the double beams, respectively. *d* (100 μm) is the thicknesses of the L-shaped beams (L_1_, L_2_, L_3_, L_4_, L_5_, and L_6_), named *d*_L1_, *d*_L2_, *d*_L3_, *d*_L4_, *d*_L5_, and *d*_L6_, respectively. *l*_2_ (2600 μm) and *w*_2_ (850 μm) are the length and the width of the two masses. Twelve piezoresistors are exploited as the sensitive elements, where the four piezoresistors (*R_x_*_1_, *R_x_*_2_, *R_x_*_3_, and *R_x_*_4_) far away from the mass were fabricated at the roots of L-shape beams (L_1_, L_2_, L_3_, and L_4_) to form the first Wheatstone bridge (*W_x_*). Meanwhile, the four piezoresistors (*R_y_*_1_, *R_y_*_2_, *R_y_*_3_, and *R_y_*_4_) close to the mass were fabricated at the roots of L-shaped beams (L_1_, L_2_, L_3_, and L_4_) to construct the second Wheatstone bridge (*W_y_*), and the other piezoresistors (*R_z_*_1_, *R_z_*_2_, *R_z_*_3_, and *R_z_*_4_) at the roots of the double beams (L_5_, L_6_) form the third Wheatstone bridge (*W_z_*) in response. *W_x_*, *W_y_*, and *W_z_* are used to measure the acceleration along *x*-axis, *y*-axis, and *z*-axis (*a_x_*, *a_y_*, and *a_z_*), respectively. Based on that, through analyzing the effect of conduction type and doping concentration on piezoresistive coefficient, the piezoresistors were selected as p-Si, and its resistivity was designed in the range of 0.01 to 0.1 Ω·cm. 

To realize a free movement of the middle double masses in the space, the back side of the chip was bonded with a glass sheet with a hole in the middle by using bonding technology, as shown in Figure 1b.

### 2.2. Theoretical Analysis of Sensing Principle 

To study the sensing principle of the chip under different accelerations, theoretical analysis was presented based on piezoresistive effect. In the condition of stress, the relative variation of the silicon piezoresistor along the same crystal orientation can be expressed as [14]

(1) $$\frac{\Delta R}{R_{0}}=\pi_{∥}\sigma_{∥}+\pi_{⊥}\sigma_{⊥},$$

where ΔR is the variation of the piezoresistor. *R*_0_ is the value of the piezoresistor without stress. $\pi_{∥}$ and $\pi_{⊥}$ are the longitudinal and the lateral piezoresistive coefficients, respectively. $\sigma_{∥}$ and $\sigma_{⊥}$ are the longitudinal and the lateral stresses, respectively.

From Equation (1), we can see that the main factors to influence ΔR include piezoresistive coefficients ($\pi_{∥}$ and $\pi_{⊥}$) and stresses ($\sigma_{∥}$ and $\sigma_{⊥}$). Due to the silicon belongs to the cubic crystal system, the piezoresistive coefficient along any crystal orientation can be expressed as [14,15]

(2) $$\left\{ \begin{array}{l} \pi_{∥}=\pi_{11}-2(\pi_{11}-\pi_{12}-\pi_{44})(l_{1}^{2}m_{1}^{2}+m_{1}^{2}n_{1}^{2}+n_{1}^{2}l_{1}^{2})\\ \pi_{⊥}=\pi_{12}+(\pi_{11}-\pi_{12}-\pi_{44})(l_{1}^{2}l_{2}^{2}+m_{1}^{2}m_{2}^{2}+n_{1}^{2}n_{2}^{2}) \end{array}\right.,$$

where $\pi_{11}$ and $\pi_{12}$ are the longitudinal and the lateral piezoresistive coefficients along the crystal axis orientation, respectively. $\pi_{44}$ is the shear piezoresistive coefficient. $l_{1}$, $m_{1}$ and $n_{1}$ are the cosine of the piezoresistor’s longitudinal orientation. $l_{2}$, $m_{2}$ and $n_{2}$ are the cosine of the piezoresistor’s lateral orientation.

As shown in Equation (2), it can be found that the piezoresistive coefficients of silicon along different orientations are different from each other. As is well-known, the piezoresistive coefficient of p-Si is better than that of n-Si. According to theoretical analysis, the $\pi_{∥}$ and $\pi_{⊥}$ on the (100) plane of p-Si are positive and negative, respectively, i.e., $\pi_{∥}$ along [011] is $\pi_{44}/2$ and $\pi_{⊥}$ along $\left[0\bar{1}1\right]$ is $-\pi_{44}/2$. Thus, it is possible to obtain a maximum piezoresistive coefficient. Based on the above analysis, the piezoresistors were designed along [011] and $\left[0\bar{1}1\right]$ orientations.

**Figure 2:**
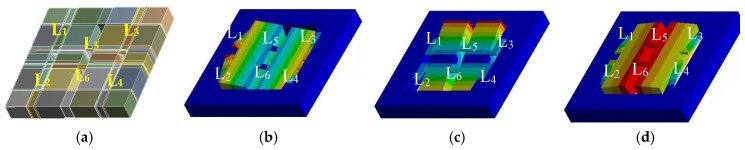
The deformation diagram of the chip under the acceleration along three-axis directions: (**a**) *a_x_* = *a_y_*= *a_z_* = 0 g; (**b**) *a* = *a_x_*; (**c**) *a* = *a_y_*; and (**d**) *a* = *a_z_*.

**Figure 3:**
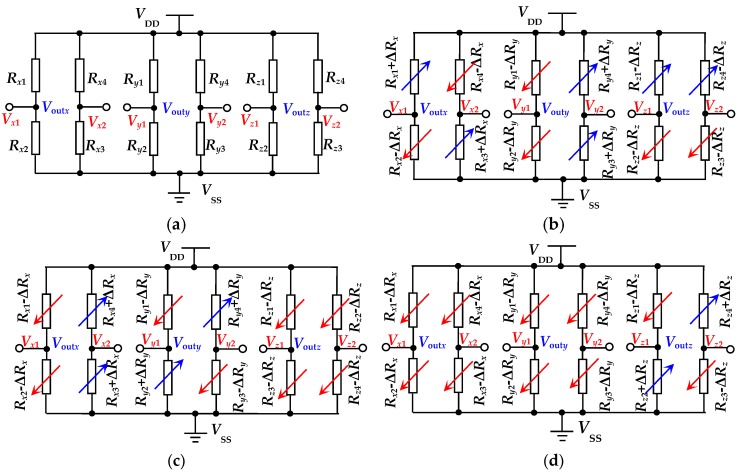
Equivalent circuit of the SOI three-axis acceleration sensor: (**a**) *a_x_* = *a_y_* = *a_z_* = 0 g; (**b**) *a* = *a_x_*; (**c**) *a* = *a_y_*; and (**d**) *a* = *a_z_*.

To analyze the working principle of the chip, a simulation model was built by using ANSYS finite element software. Based on this model, the effects of acceleration on the deformations of the L-shaped beams and the middle double beams were investigated. Figure 2 shows the deformation diagrams of the beams in the condition of *a* = 0 g, *a* = *a_x_*, *a* = *a_y_*, and *a* = *a_z_*, respectively. To further analyzing the sensing characteristics of the acceleration sensor, twelve piezoresistors on the beams are equivalent to three Wheatstone bridge circuits, with an equivalent circuit diagram under the action of *a* = 0 g, *a* = *a_x_*, *a* = *a_y_*, and *a* = *a_z_*, respectively, as shown in Figure 3.

In an ideal case, no deformation exhibits in the structure of the proposed sensor under no acceleration along *x*-axis, *y*-axis, and *z*-axis, leading to an equal resistance value of the twelve piezoresistors and no output of *V*_out*x*_, *V*_out*y*_, and *V*_out*z*_ for the three Wheatstone bridges, as shown in Figure 3a. According to Newton’s second law, the two masses would cause a displacement along *x*-axis under the action of *a_x_* when applying acceleration along *x*-axis as shown in Figure 3b. As a result, the two L-shaped beams (L_1_ and L_2_) were squeezed and the other two L-shaped beams (L_3_ and L_4_) were stretched, as shown in Figure 2b. The deformation of L-shaped beams causes a different stress distribution at the roots of beams, resulting in the increase of *R_x_*_1_, *R_x_*_3_, *R_y3_*, and *R_y_*_4_ and the decrease of *R_x_*_2_, *R_x_*_4_, *R_y_*_1_, *R_y_*_2_, *R_z_*_1_, *R_z_*_2_, *R_z_*_3_, and *R_z_*_4_ based on the elastic theory and piezoresistive effect [16,17], as shown in Figure 3b. In view of the change of *V*_out*x*_ with the external acceleration, *a_x_* can be measured. In response, L_1_ and L_3_ were squeezed and L_2_ and L_4_ were stretched under the action of *a_y_* for the chip shown in Figure 2c; the different stress distributions at the roots of beams would cause the increase of *R_x_*_3_, *R_x_*_4_, *R_y2_*, and *R_y_*_4_ and the decrease of *R_x_*_1_, *R_x_*_2_, *R_y_*_1_, *R_y_*_3_, *R_z_*_1_, *R_z_*_2_, *R_z_*_3_, and *R_z_*_4_, as shown in Figure 3c. From the change of *V*_out*y*_ with the external acceleration, *a_y_* can be measured. When *a_z_* is applied, the middle double masses would form a displacement along *z*-axis under the action of *a_z_*, as shown in Figure 2d, resulting in the four L-shaped beams (L_1_, L_2_, L_3_, and L_4_) to be squeezed or stretched at the same time, and intermediate double beams (L_5_ and L_6_) to be bent under an external force. Since the combined actions of *R_z_*_1_ and *R_z_*_3_ acting as longitudinal resistances, and *R_z_*_2_ and *R_z_*_4_ acting as lateral resistances, it is inevitable that a reduction in *R_z_*_1_ and *R_z_*_3_ as well as increase in *R_z_*_2_ and *R_z_*_4_ will occur, as shown in Figure 3d. According to the *V*_out*z*_ changes with the external acceleration, which depends on the stress distribution on double beams (L_5_ and L_6_) and the resistance changes of *z*-axis’s piezoresistors caused by the middle double beam deformations, it is possible to achieve the measurement of *a_z_*.

Based on the piezoresistive effect and the above equivalent circuit analysis, the relationship between output voltage (*V*_out*x*_, *V*_out*y*_ and *V*_out*z*_) and relative variation of piezoresistors can be expressed as Equation (3):

(3) $$\left\{ \begin{array}{l} V_{\mathrm{out}x}=V_{x1}-V_{x2}=\frac{\Delta R_{x}}{R_{0}}\cdot V_{\mathrm{DD}}\\ V_{\mathrm{out}y}=V_{y1}-V_{y2}=\frac{\Delta R_{y}}{R_{0}}\cdot V_{\mathrm{DD}}\\ V_{\mathrm{out}z}=V_{z1}-V_{z2}=\frac{\Delta R_{z}}{R_{0}}\cdot V_{\mathrm{DD}} \end{array}\right.$$

where *V*_out_*_x_*, *V*_out_*_y_*, and *V*_out_*_z_* are the output voltages of the three Wheatstone bridges along the *x*-axis, *y*-axis, and *z*-axis, respectively. *V*_DD_ is the supply voltage and *R*_0_ is the resistance value of the piezoresistor under no external acceleration. In an ideal case, Δ*R_x_*, Δ*R_y_*, and Δ*R_z_* are the variations of piezoresistors along *x*-axis (*R_x_*_1_, *R_x_*_2_, *R_x_*_3_, and *R_x_*_4_), *y*-axis (*R_y_*_1_, *R_y_*_2_, *R_y_*_3_, and *R_y_*_4_) and *z*-axis (*R_z_*_1_, *R_z_*_2_, *R_z_*_3_, and *R_z_*_4_), respectively. 

Under no accelerations along *x*-axis, *y*-axis, and *z*-axis, Δ*R_x_,* Δ*R_y_*, and Δ*R_z_* are equal to zero, resulting in no output of *V*_out*x*_, *V*_out*y*_, and *V*_out*z*_. Nevertheless, the absolute values of Δ*R_x_,* Δ*R_y_*, and Δ*R_z_* are approximately equal under the action of acceleration along *x*-axis, *y*-axis, or *z*-axis, ideally contributing to the same values of *V*_out*x*_*, V*_out*y*_, and *V*_outz_ for the proposed sensor.

Based on the above theoretical analysis, it is possible to realize the measurement of accelerations along *x*-axis, *y*-axis, and *z*-axis by using the proposed sensor. According to the definition of sensitivity and Equation (4), when applying acceleration to the sensor, the output voltages can be expressed as

(4) $$\left[\begin{array}{l} V_{\mathrm{out}x}\\ V_{\mathrm{out}y}\\ V_{\mathrm{out}z} \end{array}\right]=\left[\begin{array}{l} S_{xx}\;S_{xy}\;S_{xz}\\ S_{yx}\;S_{yy}\;S_{yz}\\ S_{zx}\;S_{zy}\;S_{zz} \end{array}\right]\left[\begin{array}{l} a_{x}\\ a_{y}\\ a_{z} \end{array}\right]$$

where *a_x_*, *a_y_*, and *a_z_* are the components of acceleration along *x*-axis, *y*-axis, and *z*-axis, respectively. *S_xx_*, *S_yy_*, and *S_zz_* are the sensitivities along *x*-axis, *y*-axis, and *z*-axis, respectively. *S_xy_* and *S_xz_* are the *x*-axis cross-axis sensitivity under the actions of *a_y_* and *a_z_*, respectively. *S_yx_* and *S_yz_* are the *y*-axis cross-axis sensitivity under the actions of *a_x_* and *a_z_*, respectively. *S_zx_* and *S_zy_* are the cross-axis sensitivity of *z*-axis under *a_x_* and *a_z_*, respectively.

### 2.3. Simulation Analysis of Sensing Principle 

**Figure 4:**
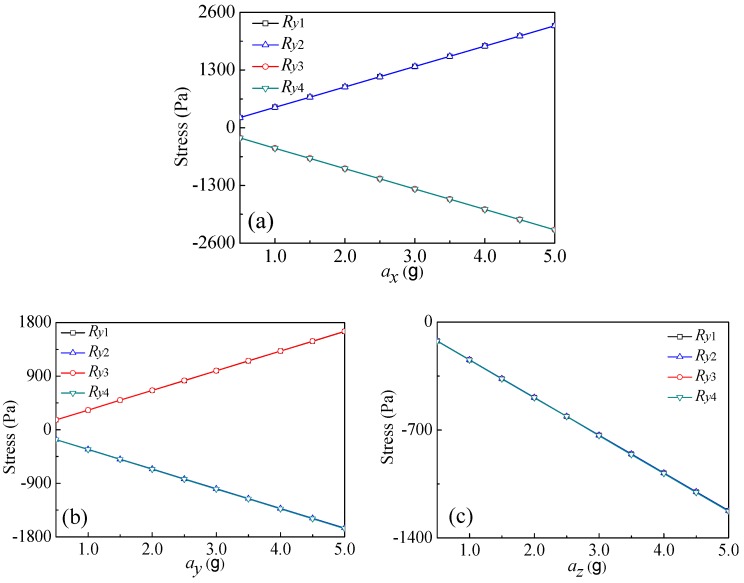
Relationship curves between the stress of L-shaped beams’ root along *y*-axis and applied acceleration: (**a**) *a* = *a_x_*; (**b**) *a* = *a_y_*; and (**c**) *a* = *a_z_*.

To analyze the sensitive characteristics and the cross-interference of sensitivity, a sensitive element simulation model of the proposed sensor was installed by using ANSYS finite element simulation software. According to Equation (1), i.e., the relative variation of piezoresistors is proportional to the stress acted, every acceleration in *x*, *y*, and *z* directions has similar behaviors associated with measurement principle, and only one of them in the three directions is needed for detailed measurement. In order to investigate the stress distributions of the four piezoresistors (*R_y_*_1_, *R_y_*_2_, *R_y_*_3_, and *R_y_*_4_) at the roots of L-shaped beams, the *y*-axis acceleration taken as example was tested by using the resulted sensitive element. Figure 4 shows the relationship curves between the max stress values along the analysis path of four piezoresistors and *a_x_*, *a_y_*, and *a_z_*, respectively. 

The analysis results indicate that when applying *a_y_* to the chip, displacements of the two masses would be caused based on Newton’s second law. As shown in Figure 4b, the roots of L_1_ and L_3_ exhibit negative stresses due to the above squeezing, in contrast, the roots of L_2_ and L_4_ display positive stresses caused by the above stretching, but both with an approximate equal absolute value of the stresses at the roots. In the ideal case, when applying *a_y_* to the chip, *V*_out*y*_ increases with the increase of *a_y_*, as shown in Figure 3c. Since L_1_ and L_2_ are squeezed and L_3_ and L_4_ are stretched under the action of *a_x_*, the resulted deformations lead to the positive stresses exhibiting at the roots of L_1_ and L_2_, also causing the equal negative stress existing at the roots of L_3_ and L_4_, as shown in Figure 4a. In the ideal condition, the *y*-axis cross-sensitivity (*S_yx_*) is ignorable under the action of *a_x_*, as shown in Figure 3b. However, all of L_1_, L_2_, L_3_, and L_4_ are stretched when applying *a_z_* to the chip, with approximately equal negative stresses at the roots of the four beams as shown in Figure 4c. From Figure 3d, the *y*-axis cross-sensitivity (*S_yz_*) can be ignored under the action of *a_z_*. 

In order to reduce the effects of different beams thicknesses on the characteristics of the proposed sensor, a SOI wafer was used as the substrate of chip and MEMS technology was utilized to realize an accurate controlling of beams thicknesses. When applying acceleration along the *z*-axis, it is possible to form constant output voltages of the proposed sensor along *x*-axis and *y*-axis directions but no cross-sensitivity (*S_xz_* and *S_yz_*) due to the same deformations of the four L-shaped beams and the equal variations of the piezoresistors along *x*-axis and *y*-axis. Similarly, the cross-sensitivity (*S_yx_*, *S_zx_*, *S_zy_*, and *S_xy_*) equals zero, and the output voltages can be expressed as Equation (5).

(5) $$\left[\begin{array}{l} V_{\mathrm{out}x}\\ V_{\mathrm{out}y}\\ V_{\mathrm{out}z} \end{array}\right]=\left[\begin{array}{l} S_{xx}\;0\;0\\ 0\;S_{yy}\;0\\ 0\;0\;S_{zz} \end{array}\right]\left[\begin{array}{l} a_{x}\\ a_{y}\\ a_{z} \end{array}\right]$$

Though above design, the proposed sensor is possible to realize the measurement of acceleration along three-axis. The simulation result indicates that it is possible to improve the sensitive characteristics and reduce the sensitivity cross-interference. 

## 3. Fabrication Technology

**Figure 5:**
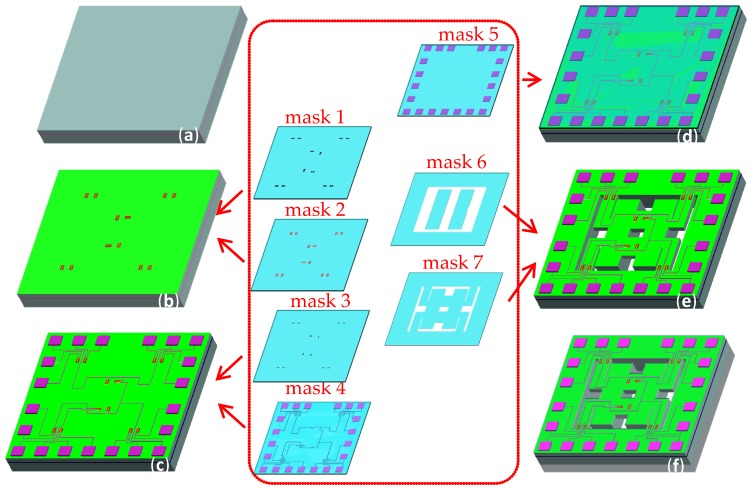
The main fabrication process of the proposed chip: (**a**) cleaning SOI wafer; (**b**) forming p- and p+ region; (**c**) forming contact hole and etching Al to form electrode; (**d**) forming pad; (**e**) etching the chip by inductively-coupled plasma (ICP) etching technology to form beam; and (**f**) bonding the back of the chip to the glass with hole.

The chip was fabricated on a SOI wafer (the device layer is <100> orientation n-Si with resistivity of 0.1 Ω·cm, and the thickness of device layer, buried oxide, and handle substrate for SOI wafer are 100 μm, 0.5 μm, and 400 μm, respectively) by using MEMS technology. The main processing steps are shown in Figure 5.

**Figure 6:**
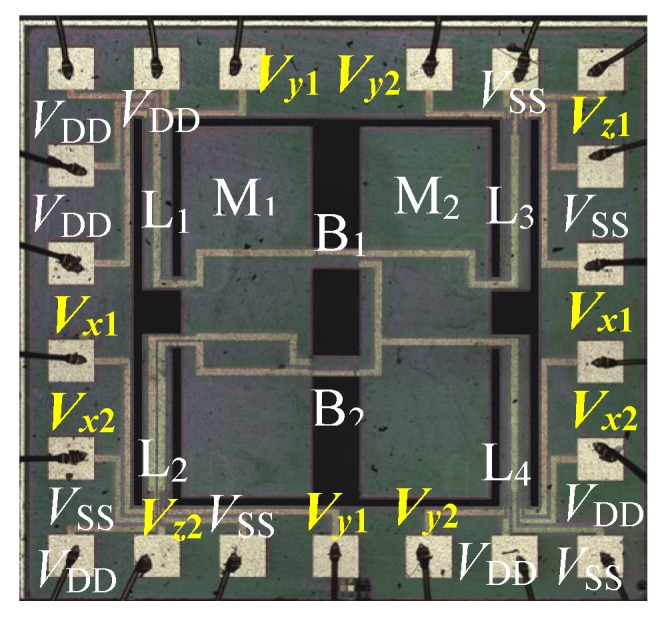
The photograph of the proposed three-axis acceleration sensor chip.

(a) Cleaning the SOI wafer by using a standard cleaning method, and first oxidation to form a SiO_2_ layer with a thickness of 50 nm by thermal oxidation method, taking as a buffer layer for the ion implantation.

(b) First photolithography to etch the SiO_2_ layer by using wet etching technology and ion implantation to perform p- region; the ion injection dose and energy were 5 × 10^13^ cm^−2^ and 60 keV, respectively. Thereafter, second photolithography to etch windows of the piezoresistors and ion implantation to perform p+ region as piezoresistors, the ion injection dose and energy of 5 × 10^15^ cm^−2^ and 60 keV, respectively, and then annealing for 30 min at 1000 °C.

(c) Etching SiO_2_ layer of 50 nm using wet etching technology and growing SiO_2_ layer of 400–500 nm as insulating layer by using plasma enhanced chemical vapor deposition (PECVD) method, third photolithography to etch the top surface to perform a contact hole and fabricate metal Al of 500 nm on the top surface of substrate by a vacuum evaporation method, fourth photolithography to form the electrodes, and then metalizing at 420 °C for 30 min to achieve ohmic contact.

(d) Growing SiO_2_ layer of 500–700 nm by using plasma enhanced chemical vapor deposition (PECVD) method to form passive layer and fifth photolithography to form pad.

(e) Etching the backside of the chip until the position of the buried oxide layer by inductively couple plasma (ICP) etching technology to form a silicon mass block. Thereafter, etching the front side of the chip until the same position of the buried oxide layer by ICP etching technology to release four L-shaped beams and the double beams.

(f) Bonding the glass sheet with the back of the chip using anodic bonding technology. Finally, the chip with an area of 4000 μm × 4000 μm was packaged on the printed circuit board (PCB) through internal lead bonding technology. As shown in Figure 6, the entire images of the chip were observed by using Olympus microscope. 

## 4. Results and Discussion

### 4.1. Testing System

**Figure 7:**
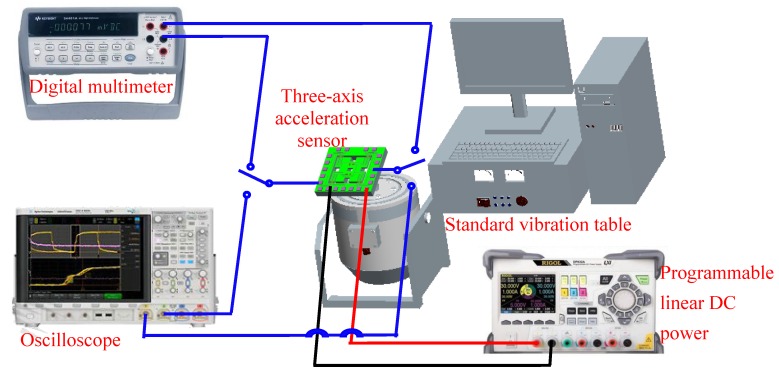
The test system of the three-axis acceleration sensor.

As shown in Figure 7, a test system of a three-axis acceleration sensor was constructed, mainly consisting of a standard vibration table (Dongling ESS-050, Suzhou, China), a digital multimeter (Agilent 34410A, Agilent Technologies, Santa Clara, CA, USA), an oscilloscope (Agilent DSO-X 4154A, Agilent Technologies), and a programmable linear direct-current power (Rigol DP832A, RIGOL Technologies. Inc., Beijing, China) in a voltage range of 0 to 30 V. The testing system can supply an excitation frequency of 5–10000 Hz and an acceleration of 0–30 g, where an accelerometer (Dytran 3120B, Dytran Instrument, Inc., Chatsworth, CA, USA) is used as a reference. At room temperature, the chip is fixed on the surface of standard vibration table. On the basis of that, some relative properties are investigated, including the resonance frequency, the sensitivity, the cross-interference of sensitivity, and so on.

### 4.2. Resonance Characteristics

**Figure 8:**
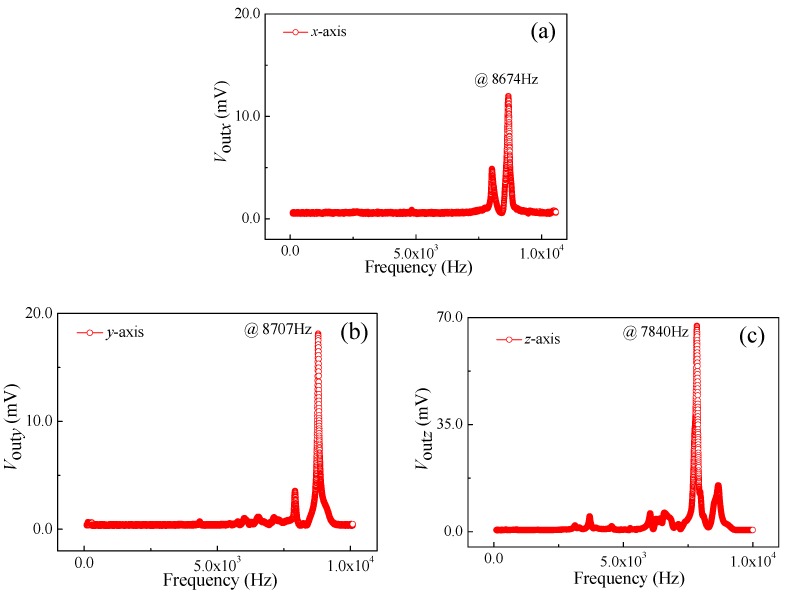
Resonance characteristics of the SOI three-axis acceleration sensor: (**a**) *a* = *a_x_*; (**b**) *a* = *a_y_*; and (**c**) *a* = *a_z_*.

Under the conditions of vibration acceleration of 3.0 g, a supply voltage of 5.0 V, as well as an excitation frequency range from 100 Hz to 10000 Hz, the relationship curves of output voltage vs. excitation frequency of *a_x_*, *a_y_*, and *a_z_* were obtained at room temperature, as shown in Figure 8. It can be seen that the output voltage changes with the increase of excitation frequency at a constant acceleration. As shown in Figure 8a, when increasing the excitation frequency along *x*-axis up to 8674 Hz, the output voltage begins to increase rapidly. Thereafter, when continually increasing the excitation frequency, the output voltage will sharply decrease. In view of the elastic theory analysis of beams, the four L-shaped beams exhibit a resonance at a frequency of 8674 Hz along the *x*-axis. By repeating the above testing process, similar resonance frequency characteristics at frequencies of 8707 Hz and 7840 Hz along the *y*-axis and *z*-axis can be observed in Figure 8b,c, respectively. It indicates that the elastic elements used to detect the acceleration along *x*-axis and *y*-axis can operate in the same way, thus achieving the approximate resonant frequency characteristics along *x*-axis and *y*-axis. Nevertheless, the different elastic structure along the *z*-axis leads to a resonant frequency of 7840 Hz lower than that of *x*-axis and *y*-axis.

**Figure 9:**
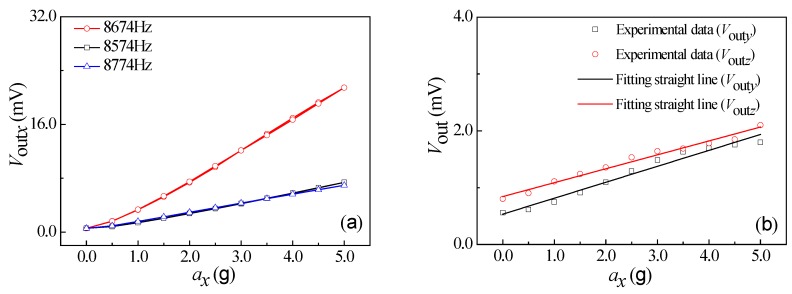
The relationship curves of output voltage and *a_x_*. (**a**) The characteristic curves of differential frequency variation. (**b**) The relationship curves between *V*_out_ along nonsensitive axis and *a_x_*.

**Figure 10:**
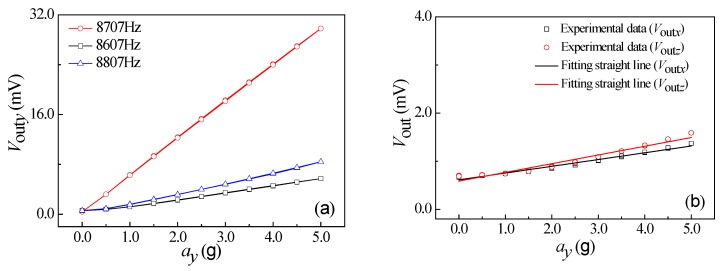
The relationship curves of output voltage and *a_y_*. (**a**) The characteristic curves of differential frequency variation. (**b**) The relationship curves between *V*_out_ along nonsensitive axis and *a_y_*.

**Figure 11:**
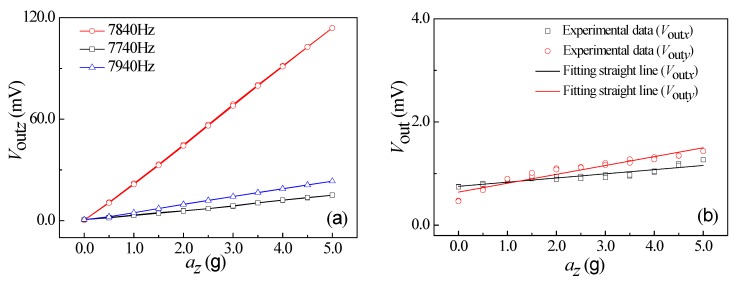
The relationship curves of output voltage and *a_z_*. (**a**) The characteristic curves of differential frequency variation. (**b**) The relationship curves between *V*_out_ along nonsensitive axis and *a_z_*.

How the acceleration influences the output voltage at the three excitation frequencies were studied at a supply voltage of 5.0 V, including a middle resonance frequency, a low resonance frequency less than 100 Hz, and a high resonance frequency more than 100 Hz. During the testing process, the external acceleration was exerted from 0 g to 5.0 g, then from 5.0 g to 0 g with a step of 0.5 g as a cycle. The test was repeated for three cycles. The relationship curves between the output voltages of the sensor and the accelerations along *x*-axis, *y*-axis, and *z*-axis are shown in Figure 9a, Figure 10a, and Figure 11a, respectively. As shown in Figure 9a, the output voltage (*V*_out*x*_) is approximately proportional to *a_x_* at a constant *V*_DD_, with a higher characteristic slope compared with the other two curves at the resonant frequency. In addition, the characteristic along *x*-axis is similar to that along *y*-axis and *z*-axis, as shown in Figure 10a and Figure 11a. In view of that, the sensitivities of three-axis acceleration sensor along *x*-axis (S*_xx_*), *y*-axis (S*_yy_*), and *z*-axis (S*_zz_*) can be calculated, that is, 4.18 mV/g, 5.88 mV/g, and 22.72 mV/g, respectively. It indicates that the proposed sensor can realize the detection of *a_x_*, *a_y_*, and *a_z_*, where S*_zz_* is higher than S*_xx_* and S*_yy_*. 

In addition, how the acceleration influences the direction of nonsensitive axes were also investigated at a supply voltage of 5.0 V. During the testing process, the acceleration changed from 0 g to 5.0 g, with a step of 0.5 g at a resonance frequency. When applying acceleration along nonsensitive axes, the output voltages of the sensor were detected. The similar cross sensitive characteristic curves along *x*-axis, *y*-axis, and *z*-axis are shown in Figure 9b, Figure 10b, and Figure 11b, respectively, where no significant variations of *V*_out*y*_ and *V*_out*z*_ occurred with changing *a_x_*. The experimental results show that the three-axis accelerometer realizes a low different cross-interference [18], i.e., the cross-interference between *z*-axis and *x*-axis as well as the *y*-axis are lower than that between *x*-axis and *y*-axis. 

### 4.3. Sensitivity Characteristics

**Figure 12:**
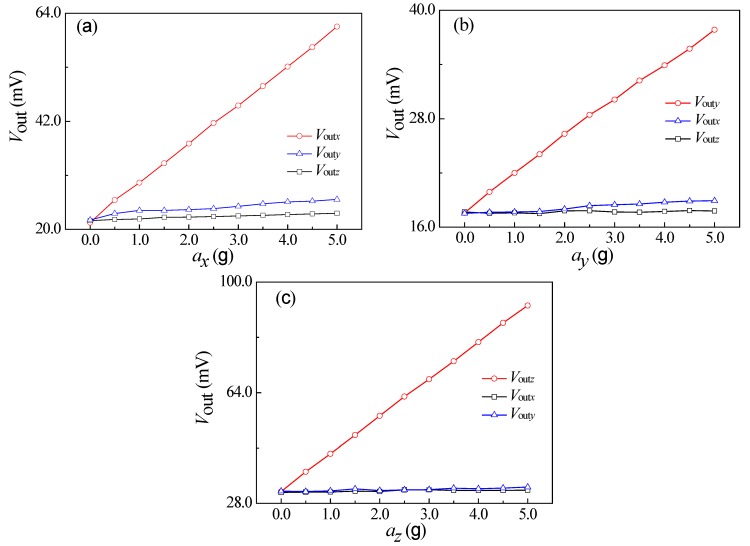
The relationship curves of output voltage and applied acceleration. (**a**) The relationship curves of output voltage and *a_x_*. (**b**) The relationship curves of output voltage and *a_y_*. (**c**) The relationship curves of output voltage and *a_z_*.

In addition, the sensitivity characteristics at the frequency of 160 Hz were investigated by varying the acceleration from 0 g to 5.0 g with a step of 0.5 g at a supply voltage of 5.0 V. Since the output signal of proposed sensor was small at a frequency of 160 Hz, the output signal was amplified by an instrumentation amplifier. In order to comparative analysis of sensitivity along sensitive axis and nonsensitive axis, we used the potentiometer to adjust the output voltages of proposed sensor without external acceleration in testing process. The relationship curves between the output voltage of proposed sensor and the external acceleration are shown in Figure 12.

Since the output voltage was small at a low frequency, it is necessary to amplify the output signals of the proposed sensor through an instrumentation amplifier, leading to an increase in zero drift. It can be seen that *a_x_* is approximately proportional to *V*_out*x*_, yet exhibits a very small effect on *V*_out*y*_ and *V*_out*z*_, as shown in Figure 12a. When applying acceleration along *y*-axis, *V*_out*y*_ is approximately proportional to *a_y_*, without significant changes of *V*_out*x*_ and *V*_out*z*_ with *a_y_*, as shown in Figure 12b. From Figure 12c, *V*_out*z*_ is approximately proportional to *a_z_*, with a constant *V*_out*x*_ and *V*_out*y*_ when changing *a_z_*. 

Based on the above data analysis, the sensitivity and the cross-sensitivity of the sensor can be calculated from Equation (4). Table 1 shows the characteristic parameters of the proposed sensor, clearly representing the sensitivities of 0.255 mV/g, 0.131 mV/g, and 0.404 mV/g prior to the amplification along *x*-axis, *y*-axis, and *z*-axis, and a minimum cross-interference of 2.2% (range of 0-5 g) along three directions. 

## 5. Conclusions

In conclusion, a SOI three-axis acceleration sensor was proposed in this work, consisting of two mass blocks, four L-shaped beams, and double beams in the middle. To detect the acceleration vector (*a_x_*, *a_y_*, and *a_z_*) along three directions, three Wheatstone bridges were constructed by designing the piezoresistors on four L-shaped beams and two intermediate beams. In order to investigate the cross-interference of sensitivity for the proposed sensor, a sensitive element simulation model was built by using ANSYS finite element software. On the basis of that, the sensor chip was fabricated and packaged on a printed circuit board by using MEMS technology and SOI as wafer. At room temperature and *V*_DD_ = 5.0 V, the sensitivities of the sensor along *x*-axis, *y*-axis, and *z*-axis are 0.255 mV/g, 0.131 mV/g, and 0.404 mV/g, respectively at an excitation frequency of 160 Hz. The thickness of the beams can be accurately controlled by using an SOI wafer as a substrate and manufacturing by MEMS technology. The proposed sensor can realize the detection of acceleration along three-axis directions, with a minimum cross-interference of 2.2% in the *x*-axis, *y*-axis, and *z*-axis directions. The study on the SOI three-axis acceleration sensor supplies an innovative and promising solution to realize the detection of three-axis acceleration, and provide a feasible method to improve sensitivity and cross-interference for future work.

## Figures and Tables

**Table 1:** The characteristic parameters of three-axis acceleration sensor.

	Characteristic Parameters	Resonant Frequency (Hz)	Bandwidth (Hz)	Sensitivity Along *x*-axis, *y*-axis and *z*-axis of Sensor at External Frequency of 160 Hz (mV/g)		
Acceleration Sensor				*a_x_*	*a_y_*	*a_z_*
Sensor along *x* axis		8674	100–7300	0.255	0.007	0.009
Sensor along *y* axis		8707	100–7500	0.027	0.131	0.018
Sensor along *z* axis		7840	100–6000	0.010	0.009	0.404
